# Study on heterogeneous OH oxidation of 3-methyltetraol sulfate in the atmosphere under high NO conditions†

**DOI:** 10.1039/d2ra02958h

**Published:** 2022-08-01

**Authors:** Chuanen Guo, Luyao Xu, Chenxi Zhang

**Affiliations:** Judicial Expertise Center, Shandong University of Political Science and Law Jinan 250014 P. R. China; Environment Research Institute, Shandong University Qingdao 266200 P. R. China; Jia Si-xie Agricultural College, Weifang University of Science and Technology Weifang 262700 P. R. China sdzhangcx@163.com

## Abstract

Organosulfates (OSs), also known as organic sulfate esters, are ubiquitous in atmospheric particles and used as secondary organic aerosol (SOA) markers. However, the chemical transformation mechanism of these OSs remains unclear. Therefore, we investigated the heterogeneous OH oxidation of 3-methyltetraol sulfate (3-MTS), which is one of the most abundant particulate organosulfates, by using quantum chemical and kinetic calculations. 3-MTS can easily undergo abstraction reaction with OH radicals, and the reaction rate constant is about 7.87 × 10^−12^ cm^3^ per molecule per s. The generated HCOOH, CH_3_COOH, HCHO, CH_3_CHO and 2-methyl-2,3-dihydroxypropionic acid are low-volatility species with increased water solubility, which are the main components of SOA. In addition, the OH radicals obtained from the reaction can continue to promote the oxidation reaction. The results of this study provide insights into the heterogeneous OH reactivity of other organosulfates in atmospheric aerosols, and it also provides a new understanding of the conversion of sulfur (S) between its organic and inorganic forms during the heterogeneous OH oxidation of organic sulfates.

## Abstract 2

The heterogeneous OH reactivity of 3-methyltetraol sulfate in atmospheric aerosols.

## Graphical abstract

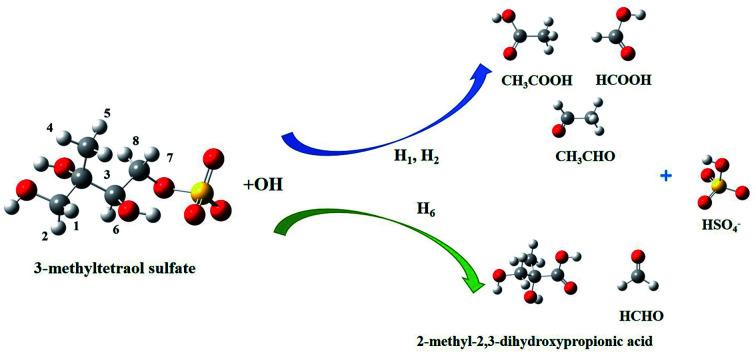

## Introduction

OSs generally refer to sulfate-containing ester compounds and their derivatives, which are an important class of SOA, accounting for about 5–30% of the total mass fraction of organic matter in PM10.^1–3^ OSs have been observed in atmospheric particles collected in rural, urban, ocean, forest and arctic regions.^4–11^ Due to the hydrophilic and hydrophobic functional groups the OSs molecules, they can contribute to climate change by affecting the hygroscopicity and light absorption of aerosols.^12,13^ Given this, it is imperative to understand the origin, formation and transformation of OSs species in the atmosphere.

OSs can be formed from the heterogeneous reaction of biogenic volatile organic compounds (BVOCs) such as isoprene, monoterpenes, sesquiterpenes, oxidized derivatives, and some chlorophyll alcohols with acidic sulfates in the atmosphere.^14–19^ In addition to natural sources, anthropogenic alkanes, polycyclic aromatic hydrocarbons, unsaturated fatty acids, and diesel fuels can also serve as precursors for OSs.^20–24^ These organic precursors can be further oxidized and then react with sulfur-containing nucleophiles to form OS.

The most abundant OSs in ambient aerosols, isoprene-derived organosulfates, are formed from the reaction of sulfates with isoprene oxidation products in the particulate phase.^25–31^ Isoprene can undergo atmospheric oxidation reaction with hydroxyl radicals (OH), and the generated OH-isoprene will be oxidized to form isoprene hydroperoxide (ISOPOOH) under low nitrogen oxide (NO_*x*_) conditions.^32,33^ ISOPOOH can further react with OH radicals to form isomeric isoprene epoxydiols (IEPOX). Under acid-catalyzed conditions, it is partitioned into sulfate aerosols through a ring-opening reaction. At the surface of the aerosol, sulfate attacks IEPOX to form a large amount of isoprene-derived OSs.^25,34–37^ Of these, the most abundant OS is methyltetraol sulfate (MTS). The data shows that, in the PM_2.5_ of downtown Atlanta and Look Rock in the United States, the proportion of organic carbon of MTS accounts for as high as 13%.^38,39^

Although the formation mechanism of OSs has been extensively studied, their chemical transformation remains unclear.^24,40–42^ These low-volatility OSs preferentially exist in the particulate phase, where they are oxidized at the aerosol surface by gas-phase oxidants, such as OH radicals, O_3_ and NO_3_ radicals.^18,43–47^ The most reactive atmospheric gas-phase radicals, especially OH radicals, readily facilitate this chemical reaction through surface interactions. For MTS, Lam *et al.* investigated the heterogeneous OH oxidation of potassium 3-MTS (C_5_H_11_SO_7_K) at 70.8% RH by using an aerosol flow tube reactor.^32^ The effective rate constant for the heterogeneous reaction is 4.74 ± 0.2 × 10^−13^ cm^3^ per molecule per s, corresponding to an atmospheric lifetime of 16.2 ± 0.3 day. Chen *et al.* investigated the oxidative aging of 2-MTS aerosols by gas-phase OH radicals at 61 ± 1% relative humidity.^48^ Kinetic measurements reveal that the effective heterogeneous OH rate constant is 4.9 ± 0.6 × 10^−13^ cm^3^ per molecule per s, with an atmospheric lifetime against OH oxidation of 16 ± 2 day, which is close to the results of 3-MTS. These suggest that MTS, as 3-MTS or 2-MTS, can exist for a longer periods in the atmosphere. In terms of the reaction mechanism, using aerosol mass spectrometry, Lam *et al.* found that OH oxidation of 3-MTS only resulted in an increase in hydrogen sulfate ions (HSO_4_^−^), while no other oxidation products were detected.^32^ Analyzing the reason, it is likely that other products are volatile and redistribute to the gas phase. Combined with aerosol phase reactions reported in previous literature, four reaction pathways with different initial hydrogen extraction points are proposed.^44,46^ Chen *et al.* paid more concerned to how 2-MTS and OH radicals were converted into OSs monitored by HILIC/ESI-HR-QTOFMS.^48^ Thus, the specific oxidation reaction mechanism between MTS and OH radicals is still unclear.

In this article, the degradation processes of the 3-MTS with OH radicals were investigated *via* the quantum chemical calculation approaches. Our results provide a pathway for atmospheric transformation of isoprene-derived organosulfates, which can better understand their potential effect on air quality and climate change.

## Computational methods

The Gaussian 09 package was chosen to perform configuration optimization and energy calculations.^49^ The M06-2X density functional method is one of the best functionals for calculating chemical thermodynamics and non-covalent interactions of main group elements.^50^ The optimal configurations of reactants, transition states, intermediates and products were obtained at the level of the 6-311++G(d,p) basis set. Frequency analysis was performed at the same level. Stable reactants, intermediates and products are free of imaginary frequencies. The transition state has one and only one imaginary frequency, and is further determined as the transition state corresponding to the reactants and products by the calculation of intrinsic reaction coordinates (IRC).^51^ All DLPNO-CCSD(T) single point energy calculations were carried out using the ORCA program (version 5.0) in conjunction with the cc-pVTZ basis set.^52,53^ In addition, the polarized continuum model (PCM) within a self-consistent reaction field (SCRF) theory was used as the model of the continuum solvent effects.^54,55^ The PCM has been proven to be flexible and accurate, in particularly, when the solute is accommodated in a cavity of realistic molecular shape and has been widely used for the study of many chemical processes.^56^

Rate constants were calculated by the KiSTelP program over a temperature range of 298 K and a pressure of 1.0 bar.^57^ The procedure is mainly based on transition state theory (TST) and Wigner tunneling correction.^58^

## Results and discussion

It is more favorable for the OH to be partially solvated at the surface rather than fully solvated in bulk water.^59^ Therefore, the first oxidation step is the abstraction reaction of 3-MTS, which exists in the droplet in its ionic form, with gas-phase OH radical on the surface of the aerosol.

### Initial reactions with OH radical

#### (A) H atom abstraction reactions

**Fig. 1:**
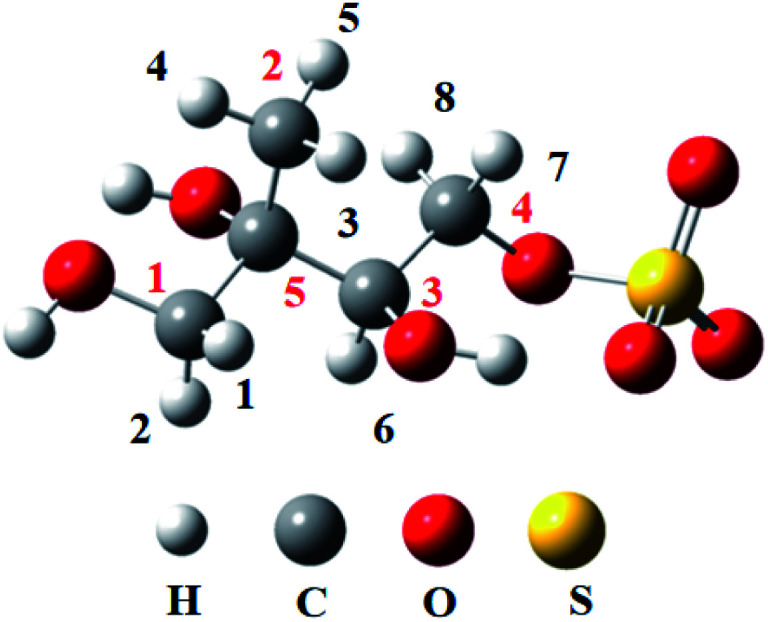
The labeled numbers in the structure of 3-MTS.

**Fig. 2:**
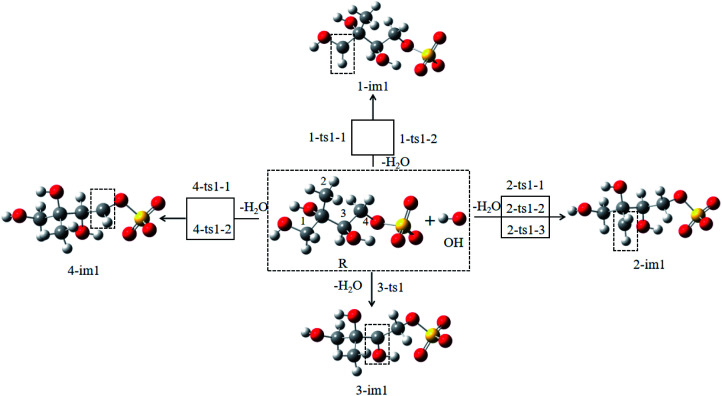
The possible reactions of 3-MTS with OH radicals.

**Fig. 3:**
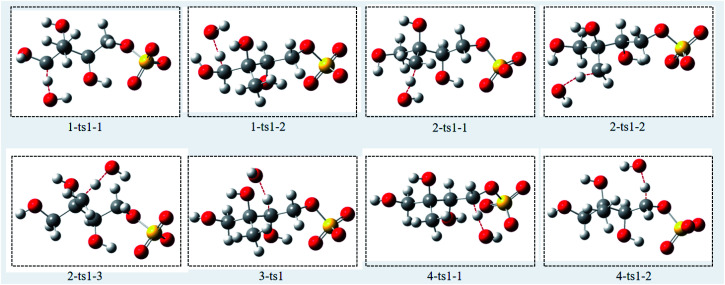
The optimized transition state structures of 3-MTS with OH radicals.

Due to the strong nucleophilicity of OH radical, the oxidation is initiated by H atom abstraction from the methyl group. For convenience, the number of H atom of the methyl group is labeled in Fig. 1. The reaction pathways of H atom abstraction are depicted in Fig. 2, and the optimized structures of the transition states involved in the reactions of 3-MTS with OH radical are depicted in Fig. 3. The rate constants *k* (cm^3^ per molecule per s) between 268 and 318 K, the relative Gibbs energy Δ*G* (kcal mol^−1^) and the branching ratios *R* (%) at 298 K in the OH oxidation of 3-MTS are shown in Table 1. Δ*G*_ts–R_ represents the Gibbs energy difference between transition state (ts) and the reactant (R), and Δ*G*_im–R_ represents the Gibbs energy difference between intermediate (im) and R.

**Table 1:** The rate constants *k* (cm^3^ per molecule per s), the relative Gibbs energy Δ*G* (kcal mol^−1^) and the branching ratios *R* (%) in the OH oxidation of 3-MTS

Reaction	*k*_268 K_	*k*_278 K_	*k*_288 K_	*k*_298 K_	*k*_308 K_	*k*_318 K_	Δ*G*_ts–R(298 K)_	Δ*G*_im–R(298 K)_	*R*_298 K_
R + OH → 1-ts1-1 → 1-im1 + H_2_O	5.13 × 10^−12^	4.54 × 10^−12^	4.06 × 10^−12^	3.67 × 10^−12^	3.34 × 10^−12^	3.07 × 10^−12^	4.61	−22.96	52.67%
R + OH → 1-ts1-2 → 1-im1 + H_2_O	5.60 × 10^−13^	5.28 × 10^−13^	5.01 × 10^−13^	4.77 × 10^−13^	4.57 × 10^−13^	4.40 × 10^−13^	5.99		
R + OH → 1-ts1-1 → 2-im1 + H_2_O	1.50 × 10^−13^	1.45 × 10^−13^	1.41 × 10^−13^	1.37 × 10^−13^	1.34 × 10^−13^	1.31 × 10^−13^	6.63	−16.22	2.05%
R + OH → 2-ts1-2 → 2-im1 + H_2_O	2.11 × 10^−14^	2.17 × 10^−14^	2.23 × 10^−14^	2.29 × 10^−14^	2.35 × 10^−14^	2.41 × 10^−14^	7.24		
R + OH → 2-ts1-3 → 2-im1 + H_2_O	1.68 × 10^−15^	1.93 × 10^−15^	2.21 × 10^−15^	2.50 × 10^−15^	2.82 × 10^−15^	3.16 × 10^−15^	11.27		
R + OH → 3-ts1 → 3-im1 + H_2_O	4.78 × 10^−12^	4.22 × 10^−12^	3.77 × 10^−12^	3.39 × 10^−12^	3.08 × 10^−12^	2.83 × 10^−12^	3.09	−25.44	43.06%
R + OH → 4-ts1-1 → 4-im1 + H_2_O	1.04 × 10^−13^	1.03 × 10^−13^	1.03 × 10^−13^	1.02 × 10^−13^	1.02 × 10^−13^	1.02 × 10^−13^	5.89	−21.22	2.22%
R + OH → 4-ts1-2 → 4-im1 + H_2_O	7.13 × 10^−14^	7.21 × 10^−14^	7.29 × 10^−14^	7.38 × 10^−14^	7.47 × 10^−14^	7.58 × 10^−14^	5.67		
Total (cm^3^ per molecule per s)	1.08 × 10^−11^	9.63 × 10^−12^	8.67 × 10^−12^	7.87 × 10^−12^	7.21 × 10^−12^	6.68 × 10^−12^			

As shown in Fig. 1, there are 8 different H atoms in 3-MTS that can undergo abstraction reactions. We are mainly divided into four types of hydrogen atoms, namely H_1_(H_2_) attached to C_1_, H_3_ (H_4_, H_5_) attached to C_2_, H_6_ attached to C_3_ and H_7_ (H_8_) attached to C_4_, and then we will discuss these four cases separately.

For the C_1_ site, OH radical can abstract two different H atoms to form the same product. Through geometry optimization and energy calculation, two transition states (1-ts1-1 and 1-ts1-2) were found in the abstraction reactions with the Gibbs energy barrier of 4.61 and 5.99 kcal mol^−1^, respectively. During the abstraction process, the H_1_ and H_2_ atom on C_1_ site start to transfer to the O atom on the OH radical, and finally the C_1_–H_1_ and C_1_–H_2_ bonds are broken with the formation of the O–H_1_ and O–H_2_ bonds, and then the 1-im1 is formed, and a H_2_O molecule is removed. The reaction releases 22.96 kcal mol^−1^ of Gibbs energy.

For the C_2_ site, H atom abstraction from the methyl group. After three different transition states (2-ts1-1, 2-ts2-2 and 2-ts2-3), the alkyl radical 2-im1 is formed. The potential Gibbs free energy barriers for the three reaction processes are 6.63, 7.24 and 11.27 kcal mol^−1^, respectively. H atom abstraction reaction from C_2_ site is exothermic by 16.22 kcal mol^−1^.

As for the C_3_ and C_4_ sites, their reaction mechanism is similar to the C_1_ and C_2_ sites. The whole H atom abstraction reactions form the C_3_ and C_4_ sites are strongly exothermic by 25.44 and 21.22 kcal mol^−1^.

The above results indicate that the H-abstraction reactions are all exothermic reactions with lower reaction Gibbs free energy barriers, which are expected to occur easily and may play an important role in the conversion of 3-MTS in the atmosphere.

#### (B) Reaction kinetic calculation

To quantitatively evaluate the contributions of the eight pathways and better understand the 3-MTS reaction with OH radical, the kinetics studies of initial reaction were carried out *via* KisThelP program in the temperature range of 268 to 318 K. The rate constant for H atom abstraction is denoted as *k*_abs_(*i*), and the total rate constant for the 3-MTS with OH reaction is labeled as *k*_total_. The branching ratio (*R*) for the *i*th entrance channels is determined as *k*_abs_(*i*)/*k*_total_.

As the results shown in Table 1, the rate constants of C_1_ site, C_2_ site, C_3_ site and C_4_ site are 4.15 × 10^−12^, 1.62 × 10^−13^, 3.39 × 10^−12^ and 1.76 × 10^−13^ cm^3^ per molecule per s at 298 K and 1 atm pressure, respectively. Thus the *k*_total_ is 7.87 × 10^−12^ cm^3^ per molecule per s, which is an order of magnitude higher than the experimental data 4.74 ± 0.2 × 10^−13^ cm^3^ per molecule per s.^32^ This may be related to the fact that DFT can generally reduce transition state energy.

The branching ratio (*R*) can be more intuitively express the contribution of each pathway. It is obvious that pathway C_1_-abs and C_3_-abs are dominant, whose *R* is around 52.67% and 43.06%. Thus, we will focus on the fate of 1-im1 and 3-im1. These two intermediates have unpaired electrons, which are highly reactive and will react rapidly with oxygen molecules to form peroxy radicals.

### Reactions of 1-im1

**Fig. 4:**
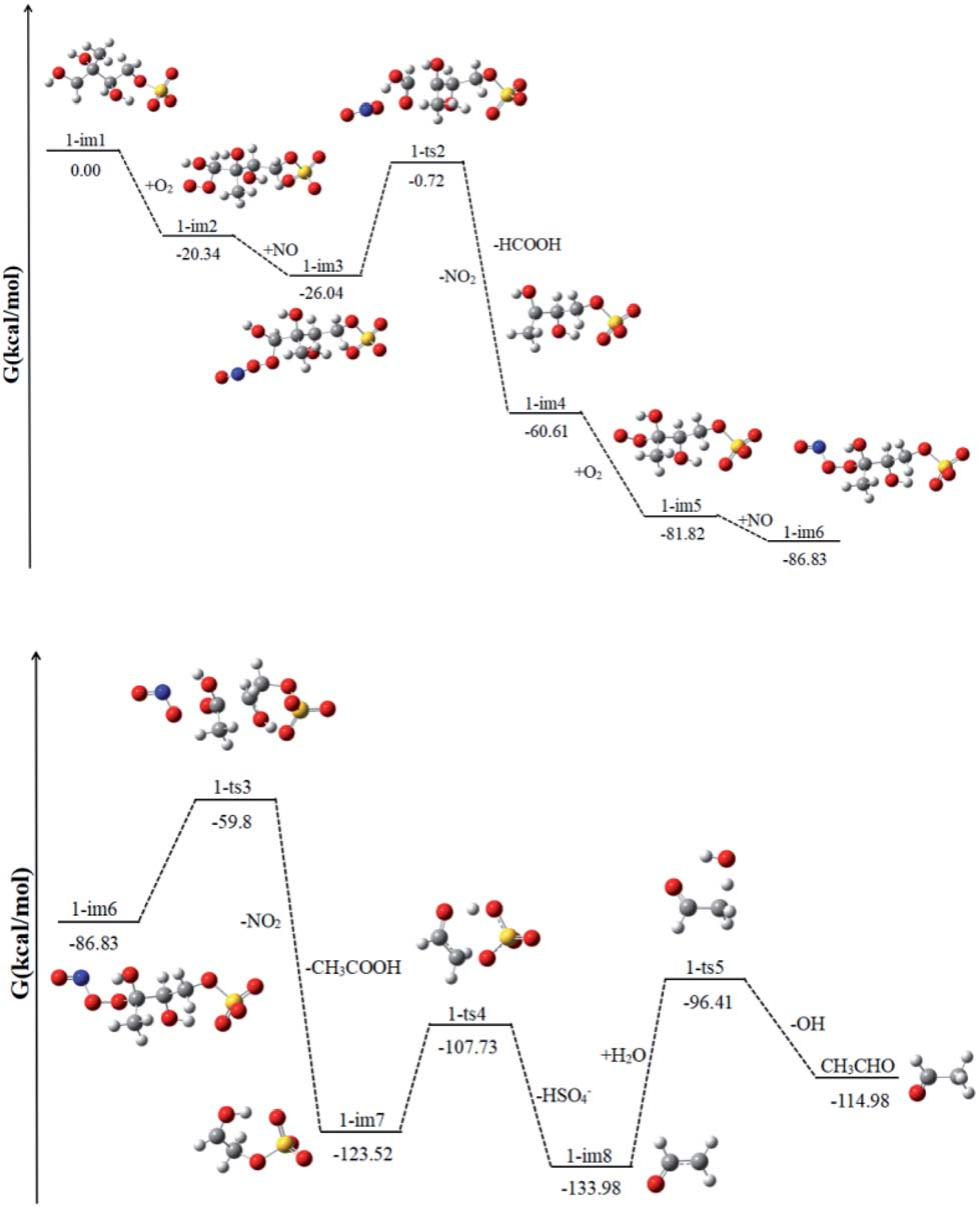
The profile of the potential energy surface for the reaction of 1-im1 at the DLPNO-CCSD(T)/cc-pVTZ//M06-2X/6-311++G(d,p) level of theory.

1-im1 can be further oxidized by the ubiquitous O_2_ in the atmosphere, which is a barrier-free reaction process. As shown in Fig. 4, it generates intermediate 1-im2, releasing Gibbs free energy of 20.34 kcal mol^−1^. Then the peroxy radical adduct 1-im2 can undergo further reaction *via* reaction with NO to form 1-im3. This process continues to release 5.70 kcal mol^−1^ of Gibbs free energy. Next, it will go through the transition state 1-ts2 and take off NO_2_. In this reaction, while the O–O bond of O_2_ is broken, the C_1_–C_5_ bond is also broken at the same time, and finally HCOOH and 1-im4 are generated. This reaction needs to cross a very high Gibbs free energy barrier, about 25.32 kcal mol^−1^, and continues to release 34.57 kcal mol^−1^ of Gibbs free energy. It is the rate determining mining step in the reaction path.

Since 1-im4 has unpaired electrons, it will continue to react with O_2_ and NO to form 1-im6, releasing 26.22 kcal mol^−1^ of Gibbs free energy. After crossing the Gibbs free energy barrier of 27.03 kcal mol^−1^, NO_2_ will be removed, and with the cleavage of the C_5_–C_3_ bond, acetic acid and 1-im7 will be obtained. SO_4_^−^ in im7 can extract a H atom from the adjacent O–H bond, while the C_4_–O cleavage occurs to form bisulfate ion (HSO_4_^−^) and ˙CH_2_CHO radicals (1-im8). 1-im8 can extract H atoms from H_2_O to generate CH_3_CHO with regenerating OH radicals. The regenerated OH radicals will initiate a new round of reactions.

The difference between our findings and the route proposed by Lam *et al.* is that in the case of high NO content, the RO_2_ radical can react with NO and the removal of NO_2_ will be accompanied by the cleavage of the C–C bond.^32^ The process can produce less volatile and more water soluble species, such as HCOOH, CH_3_CHO and CH_3_COOH, which can form SOA by nucleation, condensation, and/or partitioning between the condensed and gas phases. The production of HCOOH was also detected during the reaction of 2-MTS with OH.^48^ In addition, the produced HSO_4_^−^ has been detected by aerosol mass spectrometry.^32^ And experiments show that the HSO_4_^−^ content increases obviously with the increase of time.

### Reactions of 3-im1

**Fig. 5:**
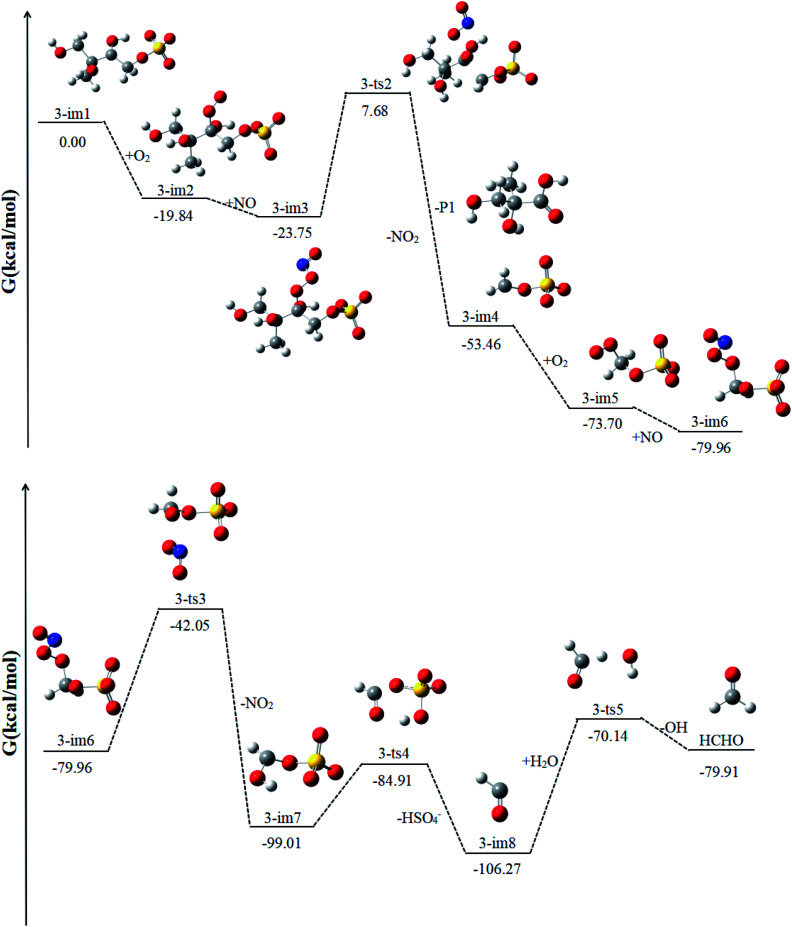
The profile of the potential energy surface for the reaction of 3-im1 at the DLPNO-CCSD(T)/cc-pVTZ//M06-2X/6-311++G(d,p) level of theory.

Similar to 1-im1, 3-im1 can undergo three elementary reactions: O_2_ addition, NO addition, NO_2_ elimination (Fig. 5). It should be pointed out that O_2_ addition and NO addition are barrier-free combination, resulting in an energy-rich intermediate (3-im3) that can be further reacted through unimolecular decomposition. The NO_2_ elimination reaction has a high potential Gibbs free energy barrier of 31.43 kcal mol^−1^. When the NO_2_ is removed, the C_3_–C_4_ bond will also be broken. This process will form the intermediate 3-im4 and P1 (2-methyl-2,3-dihydroxypropionic acid). The 3-im4 can undergo the O_2_ addition, NO addition and NO_2_ elimination to yield 3-im7. Then the 3-im7 undergo fragmentation to yield a HSO_4_^−^ and a formyl radical (CHO), which is also mentioned in the mechanism of the heterogeneous OH oxidation reaction of sodium methosulfate.^60^ The subsequent reactions of CHO radical can react with H_2_O to yield CO_2_ and OH radical. CO_2_ is volatile and can be redistributed back into the gas phase.

## Conclusions

In this paper, we applied the method of quantum chemical calculation to study the heterogeneous reaction mechanism of 3-MTS with OH radicals in the atmosphere under high NO_*x*_ conditions, and calculated the rate constants for the reaction of 3-MTS with OH radicals. The study developed a model describing the kinetics of oxidation and the formation of inorganic sulfur species. Through the research, the following meaningful conclusions have been obtained.

(1) 3-MTS can easily undergo abstraction reaction with OH radicals in the atmosphere, and its total reaction rate constant is 7.87 × 10^−12^ cm^3^ per molecule per s. In 3-MTS, there are eight C-linked H atoms that can be extracted by OH radicals to generate four intermediates. Among them, H_1_ connected to C_1_ and H_6_ connected to C_3_ are the most easily extracted.

(2) The alkyl radicals obtained by the abstraction reaction can continue to react with oxygen in the air to generate alkoxy radicals. Then under NO_*x*_-rich conditions, NO addition and NO_2_ removal reactions occur. In the process of NO_2_ removal, it is often accompanied by the breaking of C–C bonds. The generated HCOOH, CH_3_COOH, HCHO, CH_3_CHO, and 2-methyl-2,3-dihydroxypropionic acid are the main components of SOA.

(3) The CHO radical can react with H_2_O to yield CO_2_ and OH radicals. CO_2_ can be redistributed back into the gas phase. The OH radicals obtained by the reaction can continue to react with 3-MTS, thereby contributing to the occurrence of the oxidation reaction.

## Conflicts of interest

There are no conflicts to declare.

## Supplementary Material

RA-012-D2RA02958H-s001
