# Supplementary material for: Study on heterogeneous OH oxidation of 3-methyltetraol sulfate in the atmosphere under high NO conditions
Source: RSC Adv. 2022 Aug 1;12(33):21103–9. doi: 10.1039/d2ra02958h (PMC9341440; doi:10.1039/d2ra02958h)
Supplement: RA-012-D2RA02958H-s001 [file RA-012-D2RA02958H-s001.pdf]

Electronic Supplementary Material (ESI) for RSC Advances.
This journal is © The Royal Society of Chemistry 2022

# Supplementary information for

## Study on Heterogeneous OH Oxidation of 3-Methyltetraol Sulfate in the Atmosphere under High NO Conditions

Chuanen Guo[a], Luyao Xu[b], Chenxi Zhang[c,d*]

[a]Judicial Expertise Center, Shandong University of Political Science and Law, Jinan 250014, P.R.China.

[b] Environment Research Institute, Shandong University, Qingdao 266200, P.R.China.

[c] Jia Si-xie Agricultural College, Weifang University of Science and Technology, Weifang 262700, P.R.China.

[d]College of Biological and Environmental Engineering, Binzhou University, Binzhou 256600, P.R.China.

**Table S1. The relative Gibbs energy ΔG (kcal/mol) at M06-2X/6-311++G(d,p) and DLPNO-CCSD(T)/cc-pVTZ level of theory**

| Reaction | $\Delta G_{ts\text{-}R}$ （M06-2X） | $\Delta G_{ts\text{-}R}$ (DLPNO-CCSD) | $\Delta G_{im\text{-}R}$ （M06-2X） | $\Delta G_{im\text{-}R}$ (DLPNO-CCSD) |
|---|---|---|---|---|
| R+OH→1-ts1-1→1-im1+$H_2O$ | 1.02 | 4.61 | -31.02 | -22.96 |
| R+OH→1-ts1-2→1-im1+$H_2O$ | 2.31 | 5.99 | -31.02 | -22.96 |
| R+OH→1-ts1-1→2-im1+$H_2O$ | 3.00 | 6.63 | -25.27 | -16.22 |
| R+OH→2-ts1-2→2-im1+$H_2O$ | 4.02 | 7.24 | -25.27 | -16.22 |
| R+OH→2-ts1-3→2-im1+$H_2O$ | 5.33 | 11.27 | -25.27 | -16.22 |
| R+OH→3-ts1→3-im1+$H_2O$ | 0.99 | 3.09 | -33.40 | -25.44 |
| R+OH→4-ts1-1→4-im1+$H_2O$ | 3.19 | 5.89 | -28.45 | -21.22 |
| R+OH→4-ts1-2→4-im1+$H_2O$ | 3.44 | 5.67 | -28.45 | -21.22 |
| 1-im1+$O_2$→1-im2 | - | - | -26.13 | -20.34 |
| 1-im2+NO→1-im3 | - | - | -6.91 | -5.70 |
| 1-im3→1-ts2→1-im4+$NO_2$+HCOOH | 25.20 | 25.32 | -25.54 | -34.57 |
| 1-im4+$O_2$→1-im5 | - | - | -26.78 | -21.21 |
| 1-im5+NO→1-im6 | - | - | -6.77 | -5.01 |
| 1-im6→1-ts3→1-im7+$NO_2$+$CH_3COOH$ | 25.74 | 27.03 | -28.53 | -36.69 |
| 1-im7→1-ts4→1-im8+$HSO_4^-$ | 8.24 | 15.79 | -10.49 | -10.46 |
| 1-im8+$H_2O$→1-ts5→$CH_3CHO$+OH | 33.45 | 37.57 | 25.52 | 19.00 |
| 3-im1+$O_2$→3-im2 | - | - | -23.77 | -19.84 |
| 3-im2+NO→3-im3 | - | - | -6.29 | -3.91 |
| 3-im3→3-ts2→3-im4+P2+$NO_2$ | 31.07 | 31.43 | -21.51 | -29.71 |
| 3-im4+O2→3-im5 | - | - | -28.15 | -20.24 |
| 3-im5+NO→3-im6 | - | - | -8.90 | -6.26 |
| 3-im6→3-ts3→3-im7+$NO_2$ | 37.05 | 37.91 | -11.98 | -19.05 |
| 3-im7→3-ts4→3-im8+$HSO_4^-$ | 16.76 | 14.10 | -3.65 | -7.26 |
| 3-im8+$H_2O$→3-ts5→HCHO+OH | 36.00 | 36.13 | 28.56 | 26.36 |

**Table S2.** Cartesian coordinates for all the optimized structures of reactants, transition states and products

3-MTS

| | | | |
|---|---|---|---|
| C | 3.14913900 | 0.89300300 | -0.29342100 |
| H | 3.01233900 | 1.71165800 | 0.41645900 |
| H | 2.96591400 | 1.25799000 | -1.30973100 |
| C | 2.17406800 | -0.24174300 | 0.01758600 |
| C | 0.75176200 | 0.21379100 | -0.34073800 |
| H | 0.72578000 | 0.36467400 | -1.42768700 |
| C | 2.29916500 | -0.69050700 | 1.46985000 |
| H | 1.98437500 | 0.10680400 | 2.14636900 |
| H | 1.68507000 | -1.57422800 | 1.65393000 |
| H | 3.33750300 | -0.94427500 | 1.69273800 |
| O | 4.46780500 | 0.35924000 | -0.19265000 |
| H | 5.08944000 | 1.02872900 | -0.49615300 |
| O | 2.44848600 | -1.33302000 | -0.86359200 |
| H | 3.39026000 | -1.53197200 | -0.78298300 |
| O | 0.50554400 | 1.44259400 | 0.32674500 |
| H | -0.45114900 | 1.61124800 | 0.32302600 |
| O | -1.49281300 | -0.63881200 | -0.75573200 |
| S | -2.76139000 | 0.05157700 | -0.00529700 |
| O | -3.07708000 | -0.80439400 | 1.14252700 |
| O | -3.77235300 | 0.06296100 | -1.05956200 |
| O | -2.29123600 | 1.39433300 | 0.39182300 |
| C | -0.28013400 | -0.84753200 | 0.00647000 |
| H | -0.50600500 | -0.84632900 | 1.07580500 |
| H | 0.07788900 | -1.83482000 | -0.28195200 |

1-ts-1

| | | | |
|---|---|---|---|
| C | 3.01236000 | 0.18207400 | -0.67015600 |
| H | 2.95982500 | 1.27466000 | -0.31191100 |
| H | 2.83864600 | 0.18972000 | -1.74811900 |
| C | 1.93697200 | -0.64186400 | 0.05180700 |
| C | 0.55236400 | -0.21434800 | -0.46114200 |
| H | 0.51867800 | -0.45021600 | -1.53210100 |
| C | 2.06414400 | -0.53501600 | 1.56652600 |
| H | 1.86153600 | 0.48429700 | 1.90105400 |
| H | 1.36474900 | -1.21654200 | 2.05416700 |
| H | 3.07158700 | -0.81417300 | 1.88751700 |
| O | 4.31443700 | -0.29889400 | -0.45482100 |
| H | 4.53611800 | -0.21641600 | 0.48130600 |

| | | | |
|---|---|---|---|
| O | 2.07908200 | -1.99835000 | -0.36922200 |
| H | 2.98122700 | -2.27691300 | -0.16554700 |
| O | 0.41603400 | 1.18816200 | -0.27399100 |
| H | -0.52845000 | 1.42129500 | -0.24872000 |
| O | -1.77813300 | -0.87256800 | -0.55989700 |
| S | -2.95421800 | 0.10566200 | -0.00099900 |
| O | -3.32686500 | -0.40942300 | 1.31984700 |
| O | -3.98297700 | -0.03091000 | -1.02773200 |
| O | -2.33553900 | 1.44563000 | 0.05883200 |
| C | -0.56558300 | -0.98536000 | 0.22128400 |
| H | -0.73638500 | -0.61795500 | 1.23645300 |
| H | -0.33042100 | -2.04769900 | 0.25974000 |
| O | 2.73335400 | 2.63892000 | 0.26723300 |
| H | 1.77369500 | 2.44589700 | 0.27024100 |

1-ts-2

| | | | |
|---|---|---|---|
| C | 2.94474000 | 0.11240500 | 0.79435200 |
| H | 2.72121100 | -0.08518600 | 1.84184900 |
| H | 2.87709400 | 1.25681600 | 0.64162100 |
| C | 1.92083800 | -0.50942100 | -0.16059800 |
| C | 0.54721200 | 0.13159700 | 0.09471300 |
| H | 0.62257600 | 1.18346700 | -0.21087000 |
| C | 1.90372000 | -2.02545700 | -0.01699600 |
| H | 1.53958400 | -2.30729100 | 0.97246600 |
| H | 1.25841900 | -2.47589800 | -0.77421500 |
| H | 2.91289300 | -2.42340100 | -0.14243100 |
| O | 4.26238100 | -0.29479700 | 0.54566400 |
| H | 4.46637700 | -0.12520300 | -0.38403600 |
| O | 2.27714900 | -0.12442000 | -1.49258000 |
| H | 2.82868700 | -0.81178900 | -1.88544400 |
| O | 0.27980300 | 0.04524300 | 1.48552200 |
| H | -0.66694800 | 0.21045100 | 1.62943900 |
| O | -1.66787500 | 0.37720700 | -0.88324100 |
| S | -2.98026000 | 0.10361500 | 0.03828600 |
| O | -3.43981500 | -1.24976800 | -0.28954600 |
| O | -3.88019000 | 1.17475300 | -0.38322400 |
| O | -2.50728000 | 0.22788000 | 1.43204100 |
| C | -0.53970500 | -0.51817000 | -0.74583000 |
| H | -0.86448300 | -1.46700100 | -0.31071600 |
| H | -0.18298900 | -0.69276700 | -1.75975100 |
| O | 3.01390500 | 2.46762300 | -0.28262200 |
| H | 2.80629300 | 1.94447500 | -1.08043100 |

2-ts-1

| | | | |
|---|---|---|---|
| C | 3.00367000 | -0.18010300 | -0.99372800 |
| H | 2.94203500 | 0.86400900 | -1.30581500 |

| | | | |
|---|---|---|---|
| H | 2.73376700 | -0.82322100 | -1.83413800 |
| C | 2.04206800 | -0.45091000 | 0.16910900 |
| C | 0.59795300 | -0.43864700 | -0.35608800 |
| H | 0.49424700 | -1.31361600 | -1.00900400 |
| C | 2.28114400 | 0.51171400 | 1.32372900 |
| H | 2.00662200 | 1.61010600 | 1.00267600 |
| H | 1.66379500 | 0.29317400 | 2.19545900 |
| H | 3.33478100 | 0.56474100 | 1.60615900 |
| O | 4.33784100 | -0.52031300 | -0.63221100 |
| H | 4.72419400 | 0.21203800 | -0.13923300 |
| O | 2.25843100 | -1.78112900 | 0.64007900 |
| H | 3.21472600 | -1.89163100 | 0.73555100 |
| O | 0.41493400 | 0.75051300 | -1.11699400 |
| H | -0.53328100 | 0.84142300 | -1.32028500 |
| O | -1.66957900 | -1.09260100 | 0.29651800 |
| S | -2.86268800 | -0.04579800 | -0.06583600 |
| O | -3.10702000 | 0.73673500 | 1.14949700 |
| O | -3.94920800 | -0.94302100 | -0.45034500 |
| O | -2.33936600 | 0.77207800 | -1.17902900 |
| C | -0.41115300 | -0.56715300 | 0.77544500 |
| H | -0.56917800 | 0.39315600 | 1.27477700 |
| H | -0.06218900 | -1.29597000 | 1.50618700 |
| O | 1.52418000 | 2.79432100 | 0.42691000 |
| H | 1.00968200 | 2.29648100 | -0.24515300 |

2-ts-2

| | | | |
|---|---|---|---|
| C | -2.73247900 | -1.07511000 | 0.63170000 |
| H | -2.72459100 | -0.66765800 | 1.64540200 |
| H | -2.37464500 | -2.10636700 | 0.65717000 |
| C | -1.81170500 | -0.24297700 | -0.27863000 |
| C | -0.34887900 | -0.61958400 | 0.02205400 |
| H | -0.23321000 | -1.67300200 | -0.26392200 |
| C | -2.03811000 | 1.24801800 | -0.09444100 |
| H | -1.69192600 | 1.60822000 | 0.87474600 |
| H | -1.62834700 | 1.84436300 | -0.91172900 |
| H | -3.18262100 | 1.46493500 | -0.12621000 |
| O | -4.05394100 | -1.11744300 | 0.11096100 |
| H | -4.45771900 | -0.24336100 | 0.23653300 |
| O | -2.04428800 | -0.62082400 | -1.63062600 |
| H | -2.99331700 | -0.79116400 | -1.71160700 |
| O | -0.14011600 | -0.46484600 | 1.41601500 |
| H | 0.81410300 | -0.39435300 | 1.58649300 |
| O | 1.91050200 | -0.45941000 | -0.85515100 |
| S | 3.11101900 | 0.13548200 | 0.06864000 |
| O | 3.30853200 | 1.52467800 | -0.35724000 |

| | | | |
|---|---|---|---|
| O | 4.21632400 | -0.76630000 | -0.24602200 |
| O | 2.61886500 | 0.02401200 | 1.45688900 |
| C | 0.62412200 | 0.20079200 | -0.80817400 |
| H | 0.73343600 | 1.21266300 | -0.40851000 |
| H | 0.28545800 | 0.25980300 | -1.84165900 |
| O | -4.57591800 | 1.65929600 | 0.25365600 |
| H | -4.40029800 | 2.01807800 | 1.14214200 |

2-ts-3

| | | | |
|---|---|---|---|
| C | 2.99617500 | -1.36359500 | -0.17528800 |
| H | 2.84031700 | -1.70288700 | -1.19861700 |
| H | 2.73859100 | -2.17573200 | 0.51382400 |
| C | 2.08946800 | -0.15603800 | 0.13147400 |
| C | 0.64377900 | -0.65712300 | 0.26060400 |
| H | 0.60871200 | -1.29129800 | 1.15620700 |
| C | 2.26597600 | 0.90036300 | -0.94178600 |
| H | 1.75357100 | 0.64708800 | -1.87007600 |
| H | 1.82624800 | 1.91521200 | -0.57016900 |
| H | 3.31799800 | 1.12440600 | -1.12810400 |
| O | 4.36971000 | -1.03027900 | -0.05685700 |
| H | 4.55520400 | -0.85192400 | 0.87258400 |
| O | 2.42964700 | 0.35255900 | 1.42071800 |
| H | 3.17398100 | 0.95997500 | 1.33207600 |
| O | 0.34903100 | -1.42930600 | -0.89325300 |
| H | -0.61501600 | -1.47538200 | -1.00543000 |
| O | -1.58370800 | -0.04112000 | 0.98992100 |
| S | -2.86150900 | -0.13897800 | -0.01295300 |
| O | -3.12411800 | 1.22521000 | -0.48215900 |
| O | -3.89353500 | -0.69767000 | 0.85681800 |
| O | -2.42821800 | -1.04548700 | -1.09545900 |
| C | -0.34309200 | 0.47962300 | 0.45754400 |
| H | -0.52905800 | 1.00676500 | -0.48272100 |
| H | 0.02936500 | 1.18506200 | 1.19843200 |
| O | 1.34460300 | 3.23619000 | -0.28138700 |
| H | 0.46310400 | 3.10219500 | -0.67276800 |

3-ts-1

| | | | |
|---|---|---|---|
| C | 3.06652200 | -0.11150300 | 0.91380100 |
| H | 2.89282700 | -0.86506400 | 1.68504700 |
| H | 2.92106300 | 0.88658600 | 1.34027400 |
| C | 2.09088400 | -0.31068200 | -0.24611200 |
| C | 0.67859800 | 0.06810200 | 0.21709300 |
| H | 0.71119500 | 1.20290200 | 0.40511400 |
| C | 2.14986600 | -1.73700900 | -0.78409600 |
| H | 1.78335700 | -2.44268000 | -0.03568700 |
| H | 1.54484400 | -1.82872400 | -1.68805700 |

| | | | |
|---|---|---|---|
| H | 3.18126900 | -1.99477900 | -1.03250900 |
| O | 4.38186200 | -0.23349100 | 0.37946500 |
| H | 5.01012100 | 0.03920500 | 1.05595200 |
| O | 2.41244000 | 0.62251000 | -1.28080500 |
| H | 3.35892000 | 0.53712100 | -1.45747700 |
| O | 0.39277700 | -0.58907900 | 1.41962500 |
| H | -0.57334900 | -0.63897900 | 1.53897500 |
| O | -1.54414100 | 0.66283100 | -0.54755900 |
| S | -2.85244100 | -0.10523000 | 0.04906200 |
| O | -3.27063500 | -1.06603000 | -0.97505500 |
| O | -3.77826200 | 1.00111000 | 0.27251400 |
| O | -2.37556900 | -0.75595000 | 1.28658500 |
| C | -0.37899700 | -0.13006800 | -0.85572600 |
| H | -0.64924500 | -1.18623700 | -0.94576900 |
| H | -0.01107600 | 0.23076700 | -1.81497100 |
| O | 1.04437900 | 2.69036900 | 0.10003300 |
| H | 1.72508400 | 2.41236900 | -0.54405400 |

4-ts-1

| | | | |
|---|---|---|---|
| C | 3.05901600 | -0.87196900 | -0.67013800 |
| H | 2.95358400 | -0.58200100 | -1.71752500 |
| H | 2.76713400 | -1.92039200 | -0.55118000 |
| C | 2.17059500 | 0.00192600 | 0.21562100 |
| C | 0.70236700 | -0.37365000 | -0.02856100 |
| H | 0.57749000 | -1.42186100 | 0.27260500 |
| C | 2.44546200 | 1.48343700 | -0.02405100 |
| H | 2.16729400 | 1.76919300 | -1.04083800 |
| H | 1.88480800 | 2.09895800 | 0.68257600 |
| H | 3.50890600 | 1.68789200 | 0.11594000 |
| O | 4.40346400 | -0.68335100 | -0.23223800 |
| H | 4.94297300 | -1.39132000 | -0.59811800 |
| O | 2.42160600 | -0.33200800 | 1.58146200 |
| H | 3.38074000 | -0.32149100 | 1.69908900 |
| O | 0.43638100 | -0.22806500 | -1.41322200 |
| H | -0.36817600 | -0.73429500 | -1.60238000 |
| O | -1.47910700 | -0.11760800 | 1.15615600 |
| S | -2.58456500 | -0.42847800 | -0.01556000 |
| O | -2.78815200 | 0.84760400 | -0.71436000 |
| O | -3.72142600 | -0.88636400 | 0.77148400 |
| O | -1.98719700 | -1.46255500 | -0.87494900 |
| C | -0.22271900 | 0.46599200 | 0.84728600 |
| H | -0.38279000 | 1.49831700 | 0.32823500 |
| H | 0.22394800 | 0.68988500 | 1.81542700 |
| O | -0.74252400 | 2.72594600 | -0.33927700 |
| H | -1.57556000 | 2.33955900 | -0.67625400 |

4-ts-2

| | | | |
|---|---|---|---|
| C | -3.09495600 | -1.16416300 | -0.35511700 |
| H | -2.88520500 | -2.07072700 | 0.21618100 |
| H | -2.96340500 | -1.36790900 | -1.42271600 |
| C | -2.14447200 | -0.04273500 | 0.06450500 |
| C | -0.73151700 | -0.37382200 | -0.43923200 |
| H | -0.74188000 | -0.28892800 | -1.53250500 |
| C | -2.19382500 | 0.18816600 | 1.57028400 |
| H | -1.77745100 | -0.67160700 | 2.09999800 |
| H | -1.62621700 | 1.08116700 | 1.84075400 |
| H | -3.22694700 | 0.32865700 | 1.89339100 |
| O | -4.42066200 | -0.70559600 | -0.10007500 |
| H | -5.03633200 | -1.28799800 | -0.55630300 |
| O | -2.53133000 | 1.14679900 | -0.63498800 |
| H | -3.47904000 | 1.26799400 | -0.48599800 |
| O | -0.43332000 | -1.70875300 | -0.05403800 |
| H | 0.52911900 | -1.83702100 | -0.04679300 |
| O | 1.51412500 | 0.45124800 | -0.68774300 |
| S | 2.83883300 | -0.21518600 | 0.02744900 |
| O | 3.13124700 | 0.62120500 | 1.19204900 |
| O | 3.82240300 | -0.15193900 | -1.04699100 |
| O | 2.40081300 | -1.57745500 | 0.37971300 |
| C | 0.33628500 | 0.56538500 | 0.09579000 |
| H | 0.53912100 | 0.41543400 | 1.15977100 |
| H | 0.00402800 | 1.67743300 | -0.00786000 |
| O | -0.52925900 | 2.99144900 | -0.15575600 |
| H | -1.41835500 | 2.62383800 | -0.35184800 |

1-im-1

| | | | |
|---|---|---|---|
| C | 3.15738000 | 0.83722100 | -0.38377900 |
| H | 2.97781000 | 1.44990600 | -1.26202900 |
| C | 2.20898600 | -0.25833800 | -0.02199100 |
| C | 0.78114400 | 0.20932400 | -0.35134200 |
| H | 0.73619600 | 0.36854800 | -1.43678900 |
| C | 2.36975300 | -0.68853300 | 1.43305600 |
| H | 2.11829000 | 0.13284100 | 2.10644700 |
| H | 1.72455600 | -1.54148400 | 1.65208200 |
| H | 3.40349600 | -0.98553100 | 1.62118700 |
| O | 4.46708300 | 0.58871100 | -0.06112900 |
| H | 5.03452400 | 1.23161500 | -0.50220200 |
| O | 2.42429800 | -1.39652200 | -0.88784800 |
| H | 3.31172400 | -1.73144100 | -0.70994700 |
| O | 0.55335400 | 1.43295900 | 0.33058800 |
| H | -0.40216300 | 1.61016300 | 0.33407900 |
| O | -1.46923800 | -0.63580600 | -0.74813600 |

| | | | |
|---|---|---|---|
| S | -2.72804400 | 0.06668500 | 0.00705800 |
| O | -3.04513800 | -0.78456100 | 1.15815500 |
| O | -3.74419600 | 0.08407200 | -1.04226300 |
| O | -2.24509900 | 1.40626300 | 0.39931100 |
| C | -0.25241100 | -0.84736500 | 0.00677000 |
| H | -0.47110400 | -0.83883600 | 1.07761400 |
| H | 0.09774300 | -1.83752000 | -0.27909000 |
| 1-im-2 | | | |
| C | 3.16741700 | 0.86130800 | -0.33076400 |
| H | 2.96405200 | 1.89098100 | -0.06349600 |
| C | 2.20700600 | -0.23877200 | 0.01155300 |
| C | 0.78308300 | 0.21152000 | -0.35469900 |
| H | 0.75706200 | 0.34345700 | -1.44398700 |
| C | 2.33194500 | -0.64371500 | 1.48335300 |
| H | 2.03822100 | 0.18287000 | 2.13296700 |
| H | 1.70452800 | -1.51107400 | 1.70183300 |
| H | 3.36989400 | -0.90512700 | 1.70226700 |
| O | 4.47957900 | 0.45924600 | -0.30786900 |
| H | 5.05587100 | 1.22972000 | -0.37134800 |
| O | 2.46735400 | -1.37257800 | -0.82497900 |
| H | 3.34992200 | -1.69970600 | -0.61203300 |
| O | 0.53976200 | 1.45069700 | 0.29287500 |
| H | -0.41777800 | 1.61489800 | 0.30011500 |
| O | -1.46180500 | -0.63953100 | -0.75192300 |
| S | -2.73093000 | 0.05560400 | -0.00681600 |
| O | -3.05407200 | -0.79880600 | 1.14015500 |
| O | -3.73782800 | 0.07000100 | -1.06491000 |
| O | -2.25692200 | 1.39672100 | 0.39150100 |
| C | -0.25013200 | -0.84155700 | 0.01356000 |
| H | -0.47546600 | -0.81949000 | 1.08281100 |
| H | 0.10411000 | -1.83488100 | -0.25688500 |
| 2-im-1 | | | |
| C | 3.19682900 | 0.84560000 | -0.32237600 |
| H | 3.02403700 | 1.73636100 | 0.28545600 |
| H | 3.07691400 | 1.09576900 | -1.38113600 |
| C | 2.19579200 | -0.24947100 | 0.04859800 |
| C | 0.78139600 | 0.19442900 | -0.38603900 |
| H | 0.78830100 | 0.27166500 | -1.48077300 |
| C | 2.26965000 | -0.56394200 | 1.50721400 |
| H | 2.07137500 | -1.57241600 | 1.84741800 |
| H | 2.40117600 | 0.22822100 | 2.23243900 |
| O | 4.49799400 | 0.31558100 | -0.08131000 |
| H | 5.14320300 | 0.88665500 | -0.51086500 |
| O | 2.47310600 | -1.41257800 | -0.73224400 |

| | | | |
|---|---|---|---|
| H | 3.40875100 | -1.61799000 | -0.60553500 |
| O | 0.52755800 | 1.46339100 | 0.19401500 |
| H | -0.43242800 | 1.61190300 | 0.21962600 |
| O | -1.47224900 | -0.64198200 | -0.74482000 |
| S | -2.74045200 | 0.04742400 | 0.00873700 |
| O | -3.05441600 | -0.81050900 | 1.15535200 |
| O | -3.75261100 | 0.06121700 | -1.04413100 |
| O | -2.26627100 | 1.38853500 | 0.40697600 |
| C | -0.25695500 | -0.83958500 | 0.01430300 |
| H | -0.46689200 | -0.78800300 | 1.08626300 |
| H | 0.08763400 | -1.84236800 | -0.23359700 |

2-im-2

| | | | |
|---|---|---|---|
| C | 3.19681400 | 0.84559700 | -0.32239200 |
| H | 3.02401800 | 1.73636600 | 0.28542500 |
| H | 3.07689300 | 1.09574700 | -1.38115600 |
| C | 2.19577700 | -0.24947300 | 0.04860000 |
| C | 0.78138400 | 0.19442800 | -0.38603900 |
| H | 0.78828700 | 0.27167000 | -1.48077300 |
| C | 2.26962500 | -0.56391300 | 1.50722300 |
| H | 2.40093900 | 0.22829300 | 2.23243900 |
| H | 2.07161800 | -1.57243600 | 1.84743300 |
| O | 4.49797900 | 0.31558200 | -0.08132100 |
| H | 5.14318900 | 0.88666300 | -0.51086400 |
| O | 2.47309700 | -1.41259100 | -0.73222000 |
| H | 3.40874900 | -1.61797900 | -0.60552800 |
| O | 0.52755400 | 1.46338800 | 0.19402200 |
| H | -0.43243700 | 1.61189100 | 0.21965200 |
| O | -1.47225900 | -0.64201000 | -0.74481900 |
| S | -2.74043700 | 0.04742900 | 0.00873900 |
| O | -3.05443300 | -0.81051700 | 1.15533800 |
| O | -3.75258200 | 0.06125600 | -1.04414400 |
| O | -2.26621100 | 1.38852100 | 0.40698700 |
| C | -0.25696100 | -0.83959400 | 0.01430100 |
| H | -0.46690000 | -0.78800800 | 1.08626200 |
| H | 0.08763400 | -1.84237600 | -0.23359500 |

2-im-3

| | | | |
|---|---|---|---|
| C | 3.19682900 | 0.84560400 | -0.32236700 |
| H | 3.02403900 | 1.73634800 | 0.28549000 |
| H | 3.07690200 | 1.09579900 | -1.38111900 |
| C | 2.19580400 | -0.24947700 | 0.04859100 |
| C | 0.78140200 | 0.19442100 | -0.38603100 |
| H | 0.78830000 | 0.27165100 | -1.48076500 |
| C | 2.26968400 | -0.56399000 | 1.50719700 |
| H | 2.40142900 | 0.22812200 | 2.23243700 |

| | | | |
|---|---|---|---|
| H | 2.07116600 | -1.57242200 | 1.84738900 |
| O | 4.49799600 | 0.31558600 | -0.08132700 |
| H | 5.14321900 | 0.88684100 | -0.51062700 |
| O | 2.47310900 | -1.41256700 | -0.73228600 |
| H | 3.40873900 | -1.61802800 | -0.60554400 |
| O | 0.52756700 | 1.46338800 | 0.19401600 |
| H | -0.43241400 | 1.61190800 | 0.21963200 |
| O | -1.47224200 | -0.64198500 | -0.74480400 |
| S | -2.74046300 | 0.04742500 | 0.00873500 |
| O | -3.05441000 | -0.81046600 | 1.15538200 |
| O | -3.75262900 | 0.06117400 | -1.04412400 |
| O | -2.26630700 | 1.38855600 | 0.40693300 |
| C | -0.25695300 | -0.83958300 | 0.01432600 |
| H | -0.46689000 | -0.78798600 | 1.08628500 |
| H | 0.08763800 | -1.84236900 | -0.23355800 |

3-im-1

| | | | |
|---|---|---|---|
| C | 3.01218600 | 0.96337700 | -0.36272000 |
| H | 2.97962800 | 1.72191300 | 0.42311700 |
| H | 2.59957400 | 1.38010800 | -1.28690200 |
| C | 2.18430600 | -0.25520600 | 0.04700900 |
| C | 0.73320900 | 0.12472700 | 0.14275700 |
| C | 2.68436300 | -0.84009500 | 1.37289300 |
| H | 2.57739300 | -0.10260400 | 2.17177000 |
| H | 2.10152100 | -1.72738600 | 1.63009700 |
| H | 3.73696900 | -1.12232100 | 1.29458900 |
| O | 4.34820400 | 0.51559700 | -0.57317100 |
| H | 4.85038800 | 1.23796700 | -0.96395300 |
| O | 2.27003400 | -1.24308900 | -0.98336700 |
| H | 3.20528800 | -1.45829400 | -1.09186700 |
| O | 0.51114900 | 1.33390800 | 0.73872100 |
| H | -0.45325600 | 1.48008300 | 0.80213300 |
| O | -1.41708000 | -0.66365400 | -0.69728000 |
| S | -2.73189700 | 0.07828000 | -0.09814700 |
| O | -3.33174000 | -0.84853700 | 0.86708500 |
| O | -3.53393500 | 0.31616200 | -1.29602400 |
| O | -2.22187900 | 1.31114900 | 0.54067600 |
| C | -0.31925900 | -0.91827800 | 0.23686200 |
| H | -0.73544900 | -0.99406200 | 1.24867900 |
| H | 0.08142800 | -1.88730900 | -0.05123700 |

4-im-1

| | | | |
|---|---|---|---|
| C | 3.18825600 | 0.81483700 | -0.39204300 |
| H | 3.09973700 | 1.70230900 | 0.23926400 |
| H | 3.00100400 | 1.09044900 | -1.43496000 |
| C | 2.17137700 | -0.24183200 | 0.03609400 |

| | | | |
|---|---|---|---|
| C | 0.76314500 | 0.26068500 | -0.30974500 |
| H | 0.72526400 | 0.43275100 | -1.39225400 |
| C | 2.33127000 | -0.58934400 | 1.51203900 |
| H | 2.09713200 | 0.27310900 | 2.13889300 |
| H | 1.67622800 | -1.41766700 | 1.78637900 |
| H | 3.36165400 | -0.89164700 | 1.70971100 |
| O | 4.48513200 | 0.23610900 | -0.26262500 |
| H | 5.11587300 | 0.80037700 | -0.72089600 |
| O | 2.35281500 | -1.40655900 | -0.77087700 |
| H | 3.29085800 | -1.63565200 | -0.72986600 |
| O | 0.56273100 | 1.50449800 | 0.37726600 |
| H | -0.38658700 | 1.70274200 | 0.35530600 |
| O | -1.44888000 | -0.66048100 | -0.73333800 |
| S | -2.78828100 | 0.02436200 | -0.01528200 |
| O | -3.08551400 | -0.82025700 | 1.14147300 |
| O | -3.76629700 | -0.03046700 | -1.09488200 |
| O | -2.33737100 | 1.37856600 | 0.34361300 |
| C | -0.29909700 | -0.72276300 | 0.03363100 |
| H | -0.39929300 | -1.18732500 | 1.00803100 |

4-im-2

| | | | |
|---|---|---|---|
| C | 3.18826200 | 0.81483900 | -0.39203000 |
| H | 3.09971900 | 1.70231200 | 0.23927100 |
| H | 3.00103400 | 1.09044600 | -1.43495300 |
| C | 2.17137800 | -0.24183400 | 0.03608500 |
| C | 0.76315100 | 0.26068800 | -0.30976400 |
| H | 0.72527900 | 0.43276100 | -1.39227200 |
| C | 2.33126000 | -0.58936300 | 1.51202700 |
| H | 2.09713300 | 0.27308400 | 2.13889200 |
| H | 1.67620300 | -1.41767900 | 1.78635600 |
| H | 3.36163900 | -0.89168700 | 1.70969800 |
| O | 4.48514000 | 0.23612300 | -0.26257900 |
| H | 5.11587500 | 0.80035500 | -0.72090400 |
| O | 2.35282100 | -1.40655100 | -0.77089800 |
| H | 3.29085200 | -1.63568000 | -0.72983500 |
| O | 0.56273000 | 1.50449700 | 0.37725100 |
| H | -0.38659100 | 1.70272300 | 0.35532100 |
| O | -1.44888200 | -0.66044500 | -0.73336400 |
| S | -2.78828400 | 0.02436000 | -0.01527400 |
| O | -3.08549900 | -0.82030000 | 1.14145500 |
| O | -3.76630700 | -0.03043900 | -1.09486700 |
| O | -2.33738100 | 1.37855600 | 0.34366500 |
| C | -0.29909600 | -0.72275900 | 0.03360000 |
| H | -0.39929700 | -1.18733200 | 1.00799300 |

1-im2

| | | | |
|---|---|---|---|
| C | 1.66592300 | -0.77602900 | 0.05295200 |
| C | 0.31778800 | -0.17926700 | -0.38466100 |
| H | 0.29181300 | -0.22645400 | -1.48095700 |
| C | 1.76980200 | -0.99166300 | 1.55825300 |
| H | 1.52505700 | -0.07938300 | 2.10389000 |
| H | 1.07894100 | -1.78071800 | 1.85966100 |
| H | 2.78240400 | -1.30101800 | 1.82699900 |
| O | 3.99844800 | -0.61849200 | -0.42111900 |
| H | 4.63364900 | -0.23268200 | -1.03759400 |
| O | 1.75267400 | -2.00819600 | -0.64904400 |
| H | 2.60579600 | -2.41212800 | -0.44387800 |
| O | 0.26187700 | 1.16847500 | 0.04732100 |
| H | -0.66713400 | 1.45497600 | 0.05816300 |
| O | -2.02864600 | -0.74327500 | -0.64909500 |
| S | -3.17579700 | 0.22516900 | -0.01899600 |
| O | -3.62000000 | -0.41471100 | 1.22269300 |
| O | -4.17245300 | 0.25346400 | -1.08604300 |
| O | -2.50045000 | 1.51822000 | 0.21215000 |
| C | -0.84683800 | -1.00336000 | 0.14267200 |
| H | -1.04215100 | -0.78341200 | 1.19557900 |
| H | -0.64591600 | -2.06746500 | 0.02998100 |
| C | 2.81903900 | 0.10392800 | -0.46904300 |
| H | 2.62052800 | 0.51085500 | -1.46262100 |
| O | 2.95500800 | 1.25681100 | 0.42663100 |
| O | 3.76297800 | 2.13683700 | -0.07178300 |

1-im3

| | | | |
|---|---|---|---|
| C | -0.92435900 | 1.12689400 | 0.06724400 |
| C | 0.32596300 | 0.36778100 | -0.41024900 |
| H | 0.34993000 | 0.45634800 | -1.50397300 |
| C | -1.00562500 | 1.26374500 | 1.58295500 |
| H | -0.92197100 | 0.29142200 | 2.07087600 |
| H | -0.19623600 | 1.90701900 | 1.93259600 |
| H | -1.95580700 | 1.71992300 | 1.86985100 |
| O | -3.25243400 | 1.39206200 | -0.44877400 |
| H | -3.79496200 | 1.31177200 | -1.24198900 |
| O | -0.81878100 | 2.40157000 | -0.55201000 |
| H | -1.64911500 | 2.87153400 | -0.39762900 |
| O | 0.21611000 | -0.99327000 | -0.03432000 |
| H | 1.10358000 | -1.38904200 | -0.02082700 |
| O | 2.72632300 | 0.57114400 | -0.65569000 |
| S | 3.74495500 | -0.51993300 | -0.00266100 |
| O | 4.24745700 | 0.07578100 | 1.23870100 |
| O | 4.74304500 | -0.67549600 | -1.05705300 |
| O | 2.91777800 | -1.72115700 | 0.22811900 |

| | | | |
|---|---|---|---|
| C | 1.59529900 | 1.00505500 | 0.13420900 |
| H | 1.74761100 | 0.74521200 | 1.18506500 |
| H | 1.55818600 | 2.08836600 | 0.03408700 |
| C | -2.20347200 | 0.47170400 | -0.50911000 |
| H | -2.04668800 | 0.12464200 | -1.53308400 |
| O | -2.51114700 | -0.65031100 | 0.31790300 |
| O | -3.56386800 | -1.34556300 | -0.28782800 |
| N | -4.75764700 | -1.00654700 | 0.41692800 |
| O | -5.64662100 | -1.57644900 | -0.05669600 |

1-im4

| | | | |
|---|---|---|---|
| C | 2.78021200 | 0.03687400 | -0.15096300 |
| C | 1.37400700 | 0.08363500 | -0.66423000 |
| H | 1.34361600 | -0.44144600 | -1.62734700 |
| C | 3.15269300 | 0.63494400 | 1.16114500 |
| H | 2.65101200 | 1.59375400 | 1.29837900 |
| H | 2.87065100 | -0.02232800 | 1.99712900 |
| H | 4.23266400 | 0.79854600 | 1.21955900 |
| O | 3.40934700 | -1.13629200 | -0.51806100 |
| H | 4.23653100 | -1.22597500 | -0.02885000 |
| O | 1.00106300 | 1.44576600 | -0.83195000 |
| H | 0.03399600 | 1.51967300 | -0.77544300 |
| O | -0.83061100 | -0.92836700 | -0.33895800 |
| S | -2.11746000 | -0.02953700 | 0.09402600 |
| O | -2.28037600 | -0.20942900 | 1.53937000 |
| O | -3.18323300 | -0.60881900 | -0.72016000 |
| O | -1.75855700 | 1.35378500 | -0.27694700 |
| C | 0.44141000 | -0.65183700 | 0.29275000 |
| H | 0.29486000 | -0.07888800 | 1.21280100 |
| H | 0.86503100 | -1.62558500 | 0.54079000 |

1-im5

| | | | |
|---|---|---|---|
| C | 0.80436500 | 0.19503200 | -0.36550800 |
| H | 0.79737100 | 0.32475100 | -1.45478800 |
| C | 2.42225500 | -0.55715300 | 1.48574300 |
| H | 2.13969700 | 0.30883200 | 2.08353600 |
| H | 1.81627000 | -1.41594800 | 1.77923400 |
| H | 3.47391000 | -0.79241900 | 1.66081900 |
| O | 2.54371800 | -1.33151100 | -0.80895000 |
| H | 3.31201700 | -1.79153900 | -0.44565000 |
| O | 0.57580400 | 1.42870800 | 0.28670500 |
| H | -0.38221000 | 1.59432900 | 0.31251200 |
| O | -1.43676400 | -0.65381600 | -0.75652000 |
| S | -2.69695100 | 0.04925500 | 0.00464900 |
| O | -3.00830700 | -0.80461900 | 1.15408700 |
| O | -3.71285600 | 0.06823400 | -1.04339500 |

| | | | |
|---|---|---|---|
| O | -2.20789400 | 1.38637800 | 0.39657000 |
| C | -0.22507200 | -0.86629500 | -0.00393200 |
| H | -0.43747800 | -0.85604200 | 1.06847000 |
| H | 0.13164800 | -1.85470000 | -0.28962100 |
| C | 2.20648600 | -0.27758100 | 0.01868900 |
| O | 3.06020600 | 0.89336900 | -0.35637000 |
| O | 4.31756900 | 0.60458500 | -0.32198500 |
| 1-im6 | | | |
| C | 0.18101400 | 0.23059200 | -0.19657600 |
| H | 0.27653600 | 0.50889500 | -1.25352600 |
| C | 1.58461100 | -0.87510000 | 1.64614200 |
| H | 1.23018600 | -0.12381200 | 2.35159800 |
| H | 0.94976200 | -1.76043800 | 1.71017900 |
| H | 2.60458200 | -1.16259600 | 1.90641200 |
| O | 1.92857300 | -1.26339900 | -0.72292600 |
| H | 2.52005300 | -1.91470600 | -0.32430800 |
| O | -0.10925200 | 1.36865800 | 0.59188000 |
| H | -1.07301200 | 1.49611000 | 0.61756900 |
| O | -2.02793700 | -0.46638100 | -0.88732800 |
| S | -3.35851700 | 0.07282400 | -0.11516100 |
| O | -3.80265100 | -1.02117900 | 0.75325700 |
| O | -4.25279900 | 0.37822800 | -1.22810900 |
| O | -2.90290300 | 1.25707600 | 0.63993800 |
| C | -0.88963700 | -0.84503500 | -0.08535100 |
| H | -1.18946900 | -0.99430300 | 0.95546200 |
| H | -0.53043600 | -1.78784900 | -0.49431100 |
| C | 1.54444600 | -0.32615100 | 0.23717400 |
| O | 2.39920800 | 0.84260400 | 0.11817500 |
| O | 3.71354400 | 0.47570400 | 0.41455100 |
| N | 4.40148100 | 0.34050300 | -0.82459400 |
| O | 5.50610600 | 0.06420700 | -0.61227400 |
| 1-im7 | | | |
| C | -2.40013300 | 0.29656600 | -0.19098800 |
| H | -3.23396600 | 0.63569800 | -0.78742100 |
| O | -2.38776500 | -1.03924300 | 0.07337800 |
| H | -1.49962500 | -1.29632400 | 0.38703800 |
| O | -0.06463500 | 0.84535000 | -0.66359900 |
| S | 1.06509300 | -0.10369100 | 0.01004000 |
| O | 1.66641400 | 0.67342200 | 1.09983300 |
| O | 1.96402900 | -0.37959400 | -1.10968800 |
| O | 0.33181700 | -1.29137800 | 0.50008800 |
| C | -1.25635100 | 1.14236100 | 0.16680200 |
| H | -0.96384100 | 1.03045000 | 1.21601500 |
| H | -1.48401800 | 2.18720900 | -0.03124500 |

1-im8

| | | | |
|---|---|---|---|
| C | 0.12988500 | 0.40963400 | 0.00001800 |
| H | 0.19322700 | 1.50613500 | 0.00002600 |
| O | 1.16791400 | -0.26575100 | -0.00001300 |
| C | -1.16639000 | -0.17331900 | -0.00000300 |
| H | -1.26941700 | -1.25163800 | 0.00006300 |
| H | -2.04809900 | 0.45362000 | -0.00007700 |

1-ts2

| | | | |
|---|---|---|---|
| C | -0.72649500 | 1.26719900 | 0.11947800 |
| C | 0.27229000 | 0.31074500 | -0.53848100 |
| H | -0.00742900 | 0.17519600 | -1.58726900 |
| C | -0.81215800 | 1.24919200 | 1.61909800 |
| H | -0.96846500 | 0.24002900 | 1.99322900 |
| H | 0.12889500 | 1.64341600 | 2.01432300 |
| H | -1.62168100 | 1.89892300 | 1.95937900 |
| O | -3.28969100 | 1.77429300 | -0.15262500 |
| H | -3.59562800 | 1.57284500 | 0.74508300 |
| O | -0.59273800 | 2.50764800 | -0.42719200 |
| H | -1.05861200 | 3.16557900 | 0.11000800 |
| O | 0.25274200 | -0.95403200 | 0.08019800 |
| H | 0.98845300 | -1.02177500 | 0.71081700 |
| O | 2.62076400 | 0.01845600 | -1.00117600 |
| S | 3.68432500 | -0.60150400 | 0.07521300 |
| O | 4.52749000 | 0.51942800 | 0.50080100 |
| O | 4.36664500 | -1.61567700 | -0.72317200 |
| O | 2.85127700 | -1.13886900 | 1.16783400 |
| C | 1.66164600 | 0.95650000 | -0.48969500 |
| H | 1.92021200 | 1.25003300 | 0.53144800 |
| H | 1.69253800 | 1.83410900 | -1.13081500 |
| C | -2.38376400 | 0.82789200 | -0.55175400 |
| H | -2.18060100 | 0.92380500 | -1.61967300 |
| O | -2.44322400 | -0.34025800 | -0.02483600 |
| O | -3.91189300 | -1.18429800 | -0.64699600 |
| N | -4.61187900 | -1.44870800 | 0.39841800 |
| O | -5.63547800 | -2.03497900 | 0.14332200 |

1-ts3

| | | | |
|---|---|---|---|
| C | 0.10629900 | 0.40986400 | -0.75612600 |
| H | 0.51281100 | 0.55217800 | -1.75211300 |
| C | 1.15921400 | -0.09854600 | 1.70216600 |
| H | 0.96252200 | 0.95252600 | 1.90276700 |
| H | 0.31096500 | -0.71050200 | 2.00712800 |
| H | 2.04148800 | -0.41592700 | 2.26324600 |
| O | 1.63925600 | -1.62250700 | -0.06178400 |
| H | 2.00025600 | -1.70299100 | -0.95780200 |

| | | | |
|---|---|---|---|
| O | -0.29307100 | 1.57828200 | -0.21973300 |
| H | -0.96344700 | 1.43010100 | 0.47921100 |
| O | -2.16529800 | -0.25658700 | -1.10459400 |
| S | -3.27231300 | -0.00440000 | 0.08624500 |
| O | -3.63213800 | -1.33241900 | 0.58300900 |
| O | -4.32644800 | 0.70216200 | -0.62943000 |
| O | -2.55528600 | 0.80885500 | 1.08914600 |
| C | -0.89303600 | -0.75922500 | -0.68673400 |
| H | -0.96095900 | -1.19416900 | 0.31088400 |
| H | -0.59502800 | -1.52617500 | -1.39634900 |
| C | 1.47688800 | -0.29494800 | 0.24632600 |
| O | 2.25301300 | 0.54595300 | -0.35277100 |
| O | 3.90740300 | 0.48836300 | 0.41179400 |
| N | 4.67089400 | 0.02636300 | -0.51215500 |
| O | 5.82956400 | -0.04235800 | -0.17633700 |

1-ts4

| | | | |
|---|---|---|---|
| C | 2.44240600 | -0.11757900 | -0.23226000 |
| H | 3.28709700 | -0.25248500 | -0.90036600 |
| O | 2.19683400 | 1.11026900 | 0.12278100 |
| H | 1.23350000 | 1.17501300 | 0.47845800 |
| O | -0.07729300 | -0.78560800 | -0.86712600 |
| S | -1.06332600 | 0.03102300 | -0.01427300 |
| O | -1.69127900 | -0.85660500 | 0.98982000 |
| O | -2.05245100 | 0.65030200 | -0.92075000 |
| O | -0.25296300 | 1.09843800 | 0.69142500 |
| C | 1.64754300 | -1.18997800 | 0.17341000 |
| H | 1.11390400 | -1.14118100 | 1.11441400 |
| H | 1.85624100 | -2.16675000 | -0.24024800 |

1-ts5

| | | | |
|---|---|---|---|
| C | -1.15890000 | 0.06515900 | 0.00001900 |
| H | -2.21453700 | 0.38528000 | 0.00006100 |
| O | -0.88167100 | -1.11429200 | -0.00001600 |
| C | -0.15490900 | 1.16192500 | -0.00000900 |
| H | -0.23297200 | 1.77843800 | 0.89820200 |
| H | -0.23284700 | 1.77821300 | -0.89838900 |
| O | 1.91943400 | -0.25361800 | 0.00000300 |
| H | 0.92968700 | 0.67109900 | 0.00011300 |
| H | 1.33141800 | -1.03225800 | 0.00004900 |

CH3CHO

| | | | |
|---|---|---|---|
| C | 0.23474800 | 0.39888400 | -0.00002500 |
| H | 0.31789700 | 1.50473400 | 0.00002200 |
| O | 1.22597200 | -0.27825600 | -0.00000500 |
| C | -1.16469500 | -0.14715200 | -0.00003500 |
| H | -1.14849000 | -1.23553600 | -0.00020100 |

| | | | |
|---|---|---|---|
| H | -1.69908600 | 0.22345200 | -0.87901800 |
| H | -1.69841600 | 0.22300600 | 0.87959800 |

CH3COOH

| | | | |
|---|---|---|---|
| O | 0.78649900 | -1.02932300 | 0.00003800 |
| H | 1.72534200 | -0.79905600 | 0.00006400 |
| C | 0.09031500 | 0.12664300 | -0.00010300 |
| O | 0.62701000 | 1.19862700 | -0.00004200 |
| C | -1.38980900 | -0.12192800 | 0.00005200 |
| H | -1.66039600 | -0.70608400 | -0.88050300 |
| H | -1.66022600 | -0.70601600 | 0.88070600 |
| H | -1.91583700 | 0.82844100 | 0.00006900 |

HCOOH

| | | | |
|---|---|---|---|
| O | 1.11069600 | -0.08913600 | 0.00001600 |
| H | 1.05183200 | -1.05532200 | -0.00010600 |
| C | -0.13523600 | 0.39892300 | 0.00000400 |
| H | -0.10956000 | 1.49502600 | -0.00006900 |
| O | -1.12705300 | -0.26502000 | 0.00000300 |

HSO4

| | | | |
|---|---|---|---|
| H | 1.98574000 | 0.25899200 | 0.00157300 |
| O | -0.24268600 | 0.82601400 | 1.22716700 |
| S | -0.14810300 | 0.02957500 | -0.00018900 |
| O | -0.23788800 | 0.82700100 | -1.22730600 |
| O | -0.90330300 | -1.21602100 | -0.00211900 |
| O | 1.43186500 | -0.52851800 | 0.00244000 |

3-im2

| | | | |
|---|---|---|---|
| C | -3.11040800 | -0.41862000 | 0.67982900 |
| H | -2.93836600 | -0.26959400 | 1.74789000 |
| H | -2.98151600 | -1.47594600 | 0.43417200 |
| C | -2.12688400 | 0.43129400 | -0.13151600 |
| C | -2.16864600 | 1.88972500 | 0.31699300 |
| H | -1.80710000 | 1.99377500 | 1.34149700 |
| H | -1.56357100 | 2.51313200 | -0.34337600 |
| H | -3.19925800 | 2.24550700 | 0.27138600 |
| O | -4.41740300 | 0.01412700 | 0.31492600 |
| H | -5.04611400 | -0.66272500 | 0.58550700 |
| O | -2.44512800 | 0.32920300 | -1.51377200 |
| H | -3.40294100 | 0.44158500 | -1.58598400 |
| O | -0.44106000 | -0.31466000 | 1.36001600 |
| H | 0.52477500 | -0.34818400 | 1.51411900 |
| O | 1.54474500 | -0.09335200 | -0.91594200 |

| | | | |
|---|---|---|---|
| S | 2.79874800 | 0.20741000 | 0.09237400 |
| O | 3.13005000 | 1.62214400 | -0.08137100 |
| O | 3.80466600 | -0.73218400 | -0.38755700 |
| O | 2.27457500 | -0.10040600 | 1.43933100 |
| C | 0.34369100 | 0.66589100 | -0.73739400 |
| H | 0.54540000 | 1.59708400 | -0.20345800 |
| H | -0.02853200 | 0.89304400 | -1.73479100 |
| C | -0.70628100 | -0.14073400 | 0.03130000 |
| O | -0.75371100 | -1.47299000 | -0.65640700 |
| O | 0.01931800 | -2.35332900 | -0.11674900 |

3-im3

| | | | |
|---|---|---|---|
| C | 2.57826300 | -1.01910000 | 0.93492600 |
| H | 2.16503500 | -1.53318300 | 1.80494800 |
| H | 2.83728500 | 0.00132100 | 1.21109900 |
| C | 1.55966900 | -1.05771600 | -0.21956600 |
| C | 1.13053600 | -2.51216800 | -0.43337200 |
| H | 0.52907100 | -2.86553500 | 0.40713800 |
| H | 0.56124000 | -2.62181100 | -1.35702200 |
| H | 2.02171900 | -3.13698600 | -0.50872500 |
| O | 3.74238500 | -1.70058200 | 0.46526800 |
| H | 4.51081700 | -1.32173400 | 0.90277000 |
| O | 2.16137200 | -0.54480700 | -1.39952500 |
| H | 3.01886400 | -0.98455700 | -1.48255800 |
| O | 0.02161800 | -0.26680900 | 1.41447400 |
| H | -0.92440700 | -0.07776700 | 1.56416500 |
| O | -1.88846300 | 0.46039800 | -0.78870200 |
| S | -3.23042600 | 0.10839100 | 0.07816500 |
| O | -3.77919000 | -1.11932200 | -0.50112400 |
| O | -4.04001600 | 1.30531600 | -0.12216300 |
| O | -2.74325600 | -0.07238300 | 1.46074800 |
| C | -0.85519200 | -0.53182300 | -0.85513300 |
| H | -1.24315600 | -1.50982700 | -0.56779800 |
| H | -0.51847000 | -0.56348200 | -1.89053500 |
| C | 0.31960700 | -0.16314100 | 0.06216200 |
| O | 0.52220800 | 1.21811800 | -0.30089600 |
| O | 1.59582400 | 1.75318400 | 0.42151500 |
| N | 2.54890200 | 2.19365700 | -0.53135200 |
| O | 3.46866900 | 2.62031200 | 0.03181000 |

3-im4

| | | | |
|---|---|---|---|
| O | 0.98915900 | -0.74533000 | 0.15809800 |
| S | -0.46153000 | 0.04348400 | -0.01042300 |
| O | -0.39624300 | 1.16570700 | 0.92834000 |
| O | -1.39953900 | -1.01249200 | 0.35892100 |
| O | -0.51110800 | 0.44844800 | -1.41702500 |

| | | | |
|---|---|---|---|
| C | 2.13077300 | 0.00166400 | -0.04704200 |
| H | 2.11350200 | 1.04170800 | 0.25426300 |
| H | 3.02819900 | -0.59809200 | -0.03191800 |

3-im5

| | | | |
|---|---|---|---|
| O | -0.25242500 | 0.17202900 | -0.84016600 |
| S | 1.12930800 | -0.09268300 | 0.05399200 |
| O | 1.72241400 | 1.23554100 | 0.21589000 |
| O | 1.86009900 | -0.99358000 | -0.82929500 |
| O | 0.66499600 | -0.68853900 | 1.30750900 |
| C | -1.25727900 | 0.93363800 | -0.23951500 |
| H | -0.87927400 | 1.65593600 | 0.48365500 |
| H | -1.84700000 | 1.39262100 | -1.02916000 |
| O | -2.13763400 | 0.07863900 | 0.52782100 |
| O | -2.83232200 | -0.70002200 | -0.24191800 |

3-im6

| | | | |
|---|---|---|---|
| O | -0.28031400 | 0.25616600 | 0.82202000 |
| S | -1.57435600 | -0.28156500 | -0.06157700 |
| O | -2.65709700 | 0.65207100 | 0.26141100 |
| O | -1.74480100 | -1.62798200 | 0.47608300 |
| O | -1.14919400 | -0.22213700 | -1.46234400 |
| C | 0.29435700 | 1.48374600 | 0.44083800 |
| H | -0.41933000 | 2.15635100 | -0.03892400 |
| H | 0.72725000 | 1.92026100 | 1.33874900 |
| O | 1.30046500 | 1.29314000 | -0.53427600 |
| O | 2.36194900 | 0.61572800 | 0.07860900 |
| N | 2.23841800 | -0.76682400 | -0.27180400 |
| O | 3.09983100 | -1.35527200 | 0.22637300 |

3-im7

| | | | |
|---|---|---|---|
| O | -0.50880700 | 0.73744600 | -0.63907700 |
| S | 0.78298400 | -0.06514900 | 0.03897600 |
| O | 1.14708500 | 0.71228000 | 1.22277900 |
| O | 1.75555900 | -0.02924500 | -1.04628600 |
| O | 0.24521300 | -1.39838600 | 0.34675700 |
| C | -1.69154900 | 0.68511300 | 0.09081700 |
| H | -1.83196200 | -1.21696400 | 0.06082500 |
| H | -2.27052700 | 1.59636200 | 0.02008500 |
| O | -2.42354600 | -0.45305800 | -0.04035300 |

3-im8

| | | | |
|---|---|---|---|
| C | 0.06059700 | 0.58295100 | 0.00000000 |
| H | -0.84836400 | 1.23108500 | 0.00000000 |
| O | 0.06059700 | -0.59109900 | 0.00000000 |

3-ts2

| | | | |
|---|---|---|---|
| C | 2.85221900 | -0.90853000 | 0.42177700 |
| H | 2.75746300 | -0.40997400 | 1.38742000 |

| | | | |
|---|---|---|---|
| H | 3.26690200 | -0.20893000 | -0.30734800 |
| C | 1.46930200 | -1.37010100 | -0.06776900 |
| C | 0.89273900 | -2.45625900 | 0.82697700 |
| H | 0.85417700 | -2.12055400 | 1.86314400 |
| H | -0.11482300 | -2.73838600 | 0.51263100 |
| H | 1.53287000 | -3.33648800 | 0.76648000 |
| O | 3.72356200 | -2.01058500 | 0.59852300 |
| H | 3.86238200 | -2.41746200 | -0.26556200 |
| O | 1.66282000 | -1.83117800 | -1.39662600 |
| H | 1.08779400 | -2.58772400 | -1.56603300 |
| O | 0.04214200 | 0.16750700 | 1.10689500 |
| H | -0.40976900 | 1.02779100 | 1.08999000 |
| O | -1.82928300 | 0.38633600 | -0.98854900 |
| S | -2.98409800 | 0.00680700 | 0.20078800 |
| O | -2.66368900 | -1.35740000 | 0.60751100 |
| O | -4.22534800 | 0.16372200 | -0.54543100 |
| O | -2.74622200 | 1.03427200 | 1.20993700 |
| C | -0.80410700 | -0.45776000 | -1.26472000 |
| H | -0.96245500 | -1.48977700 | -0.94244000 |
| H | -0.46504700 | -0.35454900 | -2.28717600 |
| C | 0.55096800 | -0.12983800 | -0.11266500 |
| O | 0.82597400 | 0.85321500 | -0.90965000 |
| O | 2.18501500 | 1.87666400 | -0.21597400 |
| N | 1.64536000 | 3.03254700 | -0.09401000 |
| O | 2.40650500 | 3.87172700 | 0.32995700 |

3-ts3

| | | | |
|---|---|---|---|
| O | -0.98008000 | 0.93575800 | 0.80746000 |
| S | -1.51779000 | -0.46509100 | -0.06051900 |
| O | -1.71954400 | 0.05186400 | -1.40751200 |
| O | -2.72030800 | -0.75872600 | 0.69680600 |
| O | -0.42555900 | -1.41165600 | 0.09105300 |
| C | -0.05933300 | 1.73907200 | 0.27827200 |
| H | -0.22842500 | 2.11437700 | -0.90769500 |
| H | 0.11754200 | 2.63595700 | 0.87459900 |
| O | 0.85944900 | 1.29621300 | -0.52856300 |
| O | 2.16120000 | 0.39244400 | 0.46188900 |
| N | 2.68177200 | -0.42535400 | -0.36246700 |
| O | 3.57223100 | -1.10162700 | 0.11249600 |

3-ts4

| | | | |
|---|---|---|---|
| O | 0.38908500 | -0.94410600 | -0.53307400 |
| S | -0.73126800 | -0.04298000 | 0.03337900 |
| O | -0.85825000 | -0.22339300 | 1.46886900 |
| O | -1.93013400 | -0.20319600 | -0.75973200 |
| O | -0.17980100 | 1.41441700 | -0.25033400 |

| | | | |
|---|---|---|---|
| C | 1.89199100 | -0.66155700 | 0.08032500 |
| H | 0.82148300 | 1.37436200 | -0.11492300 |
| H | 2.43342100 | -1.46467300 | -0.42937200 |
| O | 2.21578000 | 0.54969600 | 0.01530700 |

3-ts5

| | | | |
|---|---|---|---|
| C | -0.76245200 | 0.49002200 | 0.00001000 |
| H | -1.15754900 | 1.51991200 | 0.00010300 |
| O | -1.45658000 | -0.48180600 | -0.00000900 |
| O | 1.90875600 | -0.00611600 | -0.00002300 |
| H | 1.73566900 | -0.96481200 | 0.00015900 |
| H | 0.37918300 | 0.40814600 | -0.00006800 |

HCHO

| | | | |
|---|---|---|---|
| C | -0.52559800 | -0.00001200 | 0.00000800 |
| H | -1.10704800 | -0.93961400 | -0.00001600 |
| O | 0.67100300 | 0.00002500 | -0.00000200 |
| H | -1.10738600 | 0.93948300 | -0.00001600 |

P1

| | | | |
|---|---|---|---|
| C | 1.07352600 | 0.78339200 | -0.58560700 |
| H | 0.88412500 | 1.78347500 | -0.19816900 |
| H | 0.83038100 | 0.76008500 | -1.65353300 |
| C | 0.18980600 | -0.23150500 | 0.14910400 |
| C | 0.39531400 | -0.19177600 | 1.66201300 |
| H | 0.15200800 | 0.79411000 | 2.06003900 |
| H | -0.24228900 | -0.93717200 | 2.14400800 |
| H | 1.43777600 | -0.42134100 | 1.88482300 |
| O | 2.44708600 | 0.50844400 | -0.37988500 |
| H | 2.62766700 | -0.37518700 | -0.72306700 |
| O | 0.53239200 | -1.49686600 | -0.38280700 |
| H | 0.05151400 | -2.18280600 | 0.09791000 |
| O | -1.61134500 | 1.32185500 | 0.11066400 |
| H | -2.55424200 | 1.45574400 | -0.08224000 |
| C | -1.27526000 | 0.06041200 | -0.16178500 |
| O | -2.05404000 | -0.75843900 | -0.58698700 |
